# Achieving the Millennium Development Goals for Health and Nutrition in Bangladesh: Key Issues and Interventions—An Introduction

**DOI:** 10.3329/jhpn.v26i3.1893

**Published:** 2008-09

**Authors:** David A. Sack

**Affiliations:** ICDDR, B, Mohakhali, Dhaka 1212, Bangladesh

Newcomers to Bangladesh tend to consider this to be a small country because of its small geographic size. However, public-health professionals are less focused on the size of the landmass than they are on the size of the population. Geographic and environmental features shown on maps do influence the health of the population; this is especially true in Bangladesh where the density of population is far higher than any other country that is not a city state. This high density of population, along with poverty and the estuarine environment, contributes significantly to health issues for Bangladesh. However, typical maps do not reveal many features needed to understand the health issues of the country.

## BANGLADESH IS A VERY LARGE COUNTRY

**Fig. 1:**
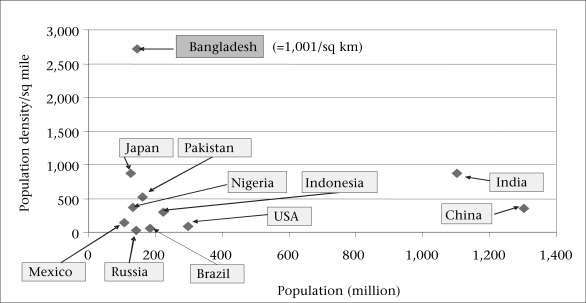
‘Mega’ - countries with population of > 100 million

Among the mega-countries, Bangladesh stands out in terms of the density of population (Fig. 1). As opposed to other countries with a population exceeding 100 million, the density of population in Bangladesh is more than twice the density of other populous countries, and the population continues to grow. Bangladesh is only half way up the population curve such that, during the next 50 years, the difference in density between Bangladesh and other countries will widen even further. Thus, the density of population, as well as poverty, and the rapid urbanization of the country are major constraints for Bangladesh while it attempts to achieve the Millennium Development Goals (MDGs). Hopefully, the fertility rate will continue to fall to levels less than needed for replacement, since this will ease one of these constraints.

Cartograms are one way to express issues of population and health. These are not accurate in geographical terms, but using computer-generated illustrations, they highlight specific attributes. Different methods can be used for preparing cartograms. The method used here preserves the general features of each country, but adjusts the area of the countries in proportion to the selected feature. The resulting images, shown on the next three pages, help illustrate the fact that Bangladesh is not at all a small country when viewed from the standpoint of the population size or magnitude of the health issues facing health planners.

**Fig. 2:**
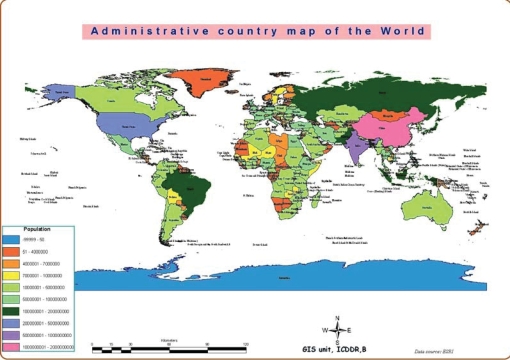
Administrative country map of the world

**Fig. 3:**
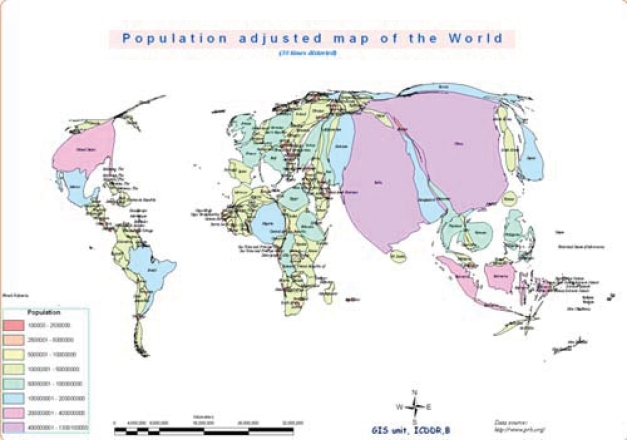
Population-adjusted map of the world

The first map (Fig. 2), which serves as the starting point, is a typical administrative world map and is a familiar map. The second cartogram (Fig. 3) looks distorted because it adjusts the area of each country in proportion to the population of that country. Bangladesh, barely visible in the first map, grows considerably in the second, relative to other countries.

**Fig. 4:**
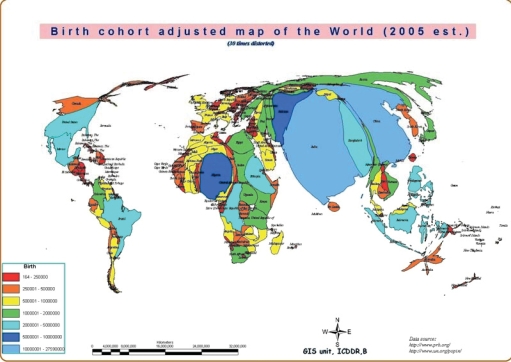
Birth cohort-adjusted map of the World (2005 estimate.)

**Fig. 5:**
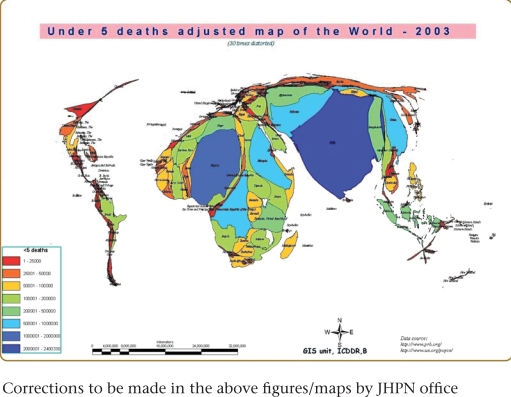
Under-five deaths-adjusted map of the world—2003

The third cartogram (Fig. 4) adjusts the areas of the countries again, but in proportion to the birth cohort. Since the MDG 4 and 5 target children and mothers, this projection illustrates where babies are being born and where pregnant mothers require services. From a global perspective, if MDG 4 and 5 are to be achieved, resources are needed in the largest countries of this cartogram. The last cartogram (Fig. 5) adjusts the areas in proportion to the annual number of deaths of children aged less than five years (under-five deaths). MDG 4 specifically addresses the reduction in under-five deaths, so achieving this goal on a global basis illustrates where additional resources are needed.

The cartograms illustrate the fact that Bangladesh is not a small country but is one where many people live, where many pregnant women need care, where children are being born, and where too many are dying. Among the countries of the world, Bangladesh, with about 340,000 childhood deaths per year, ranks number seven. Other countries with more childhood deaths are: India, Nigeria, China, Pakistan, DR Congo, and Ethiopia. Afghanistan, Tanzania, and Indonesia are in the list of 10 countries with the most number of childhood deaths.

The papers that follow describe some health issues facing Bangladesh but focus on those issues that are especially relevant to achieving the MDGs, including child health, maternal health, poverty and nutrition, and infectious diseases. In some respects, improvements in some health indicators have been remarkable, especially in reducing fertility, reducing under-five mortality, providing vaccines to children and mothers, reducing vitamin A deficiency, and others. The country is still lagging behind in other areas and will need to make significant improvements if it is to reach the MDGs which it has set.

## TRENDS AND PLATEAUS

In reviewing the trendlines towards the MDGs, there has been a tendency among many public-health professionals to think that the trends established from the past are likely to continue into the future. This concept suggests that we are either ‘on-track’ to meet the MDG or ‘not on track’. This concept seems to assume that general forces will influence the indicators as they have in the past and that the trendline will continue into the future. An improving trendline is encouraging, but as seen with plateaus in fertility and child survival, a continually-improving trend should not be assumed.

A public-health indicator often improves because of specific interventions and not because of general forces. An example is that of the reductions in neonatal mortality. Prior to initiating primary health services, neonatal mortality was very high, and it was possible to reduce this high rate very quickly by providing tetanus toxoid vaccine to pregnant mothers. This single intervention reduced neonatal mortality greatly, but in proportion to the contribution from this single disease. Without further interventions, the tetanus toxoid programme will simply re-establish a neonatal mortality rate at a somewhat lower plateau, but this single intervention does not establish an improving trendline. If one wants to reduce neonatal mortality further, one needs to introduce other interventions that address other causes of neonatal deaths. A ‘comprehensive neonatal intervention’ might result in a downward trend in neonatal mortality, but this downward trend, in fact, results from a series of stepwise improvements which have resulted from specific interventions.

Planners should not, thus, be surprised that there have been plateaus in the indicators for Bangladesh. These have been common for the population programme in which total fertility rates have come down from nearly 7 to about 3, but then have seemed to be resistant to drop further. Similarly, the infant mortality rate has come down significantly, but now has tended to plateau. In each case, the levels were very high, interventions were introduced, and the resulting improvement was observed. Further improvements will require further interventions while maintaining interventions that were previously effective. There is no reason to expect further improvements from the same interventions—the results from these interventions have already been realized.

A corollary of this observation is that the beneficial effect of a previously-implemented intervention should not be taken for granted. If a tetanus vaccination programme were to be stopped, neonatal mortality would again rise to its previous high plateau. Similarly, the improvements seen with vitamin A, or with vaccination programmes (EPI), or other programmes must be sustained, and the new interventions should be added onto the existing ones. Switching to new programmes, while allowing old programmes to deteriorate would be counter-productive.

## ACCESS OR CONNECTIONS?

In a discussion of health systems and equity, *access* is often a key issue. Persons, regardless of their socioeconomic status, should have access to healthcare, and one of the goals of an equitable health system is to assure this access. The concept of *connections* to the health system is an issue which may neither have been sufficiently stressed nor studied. Families continue to use village doctors for serious illnesses even when *access* to modern facilities is available. They may feel a connection to their village doctor, but not to the district or subdistrict health facili- ty. There are many factors that can facilitate this connection or, alternatively, impose constraints to the connection. These constraining factors include poor quality of care, costs, geographic distance, and social distance.

Just as patients need to feel a connection to their providers, the different levels of providers need to be connected. A community-based programme needs connections to facilities, and the primary health clinic needs connections to the hospital. The community health worker looses credibility if she is not connected to the higher-level facility to obtain the needed services for her clients. Primary health clinics are instructed to refer complicated and serious patients to a hospital, but generally the clinic has no connection to the hospital and can only recommend referral. Most often, the referred patient does not go, or is refused treatment because of lack of bed space or because of unofficial or official payments. Even wealthy patients need to be introduced to a person in authority to receive care—these patients are said to have ‘connections’, and these are known to be critical, especially in times of emergency. Unfortunately, a study of how to facilitate these connections has not been undertaken to minimize the requirement for these connections.

A lesson from the ICDDR, B hospital experience is that patients do not require a connection to seek care from this hospital. All patients are seen immediately and are treated effectively, regardless of their socioeconomic status. There are no barriers to effective treatment. What is not clear is how to institute this ‘no-connection needed’ policy for other health facilities.

Improvements in health services and health indicators will almost certainly require an improvement in the connections between families and the system and among the various components of the health system. Without the connections, ‘access’ to healthcare remains hypothetical, but not real.

## MAJOR LESSONS FROM THIS REVIEW

Each paper in this volume has important lessons, and this introduction cannot highlight all of them. A few themes, however, emerge.

For child health, to achieve success for MDG 4, we must reduce neonatal mortality, improve nutrition during the early months, prevent and treat the most common life-threatening infectious diseases, and develop strategies for reducing the incidence of drowning. The interventions needed include a package of services in the community to make deliveries safe for both mother and child and to prevent and manage any complications of the delivery or any illnesses that occur in the first few days of life of a newborn. New efforts are needed to improve rates of exclusive breastfeeding and to introduce proper complementary feeding. While maintaining the gains made with current vaccines and vitamin A programmes, new vaccines for pneumonia and diarrhoea are needed, and renewed efforts are needed to manage pneumonia better. Implementation of zinc as treatment for every episode of diarrhoea will be a critical new programme for the nation, and Bangladesh has the opportunity to be a leader with this new strategy. The health system needs to be improved with continued expansion of the Integrated Management of Childhood Illness (IMCI) strategy for common illnesses of children; however, this system needs continued evaluation and refinement to provide the most effective package of services that are acceptable to parents. The providers of IMCI need to be empowered to provide many services in the community and to be connected to effective higher-level care.

The programme for neonatal health will be the most important one in terms of reaching MDG 4. This may also be the most difficult programme because it is new. There are currently no similar programmes being implemented at scale in the government or NGO systems, and new skills and supervisory structures will be needed. A model of such a community-based approach is being field-tested in Sylhet and Tangail districts, and it is now known that this approach is able to reduce the number of newborn deaths significantly. Clearly, this model is only the start. Besides implementing known interventions into the new system, additional research is needed to develop community-based strategies against specific causes of neonatal deaths, for example birth asphyxia or neonatal infection.

Achieving MDG 5 (reducing maternal mortality) will be difficult unless some basic changes are made in the strategy for providing delivery-care for Bangladesh. Although the maternal mortality ratio has reduced somewhat in Bangladesh, especially in the Matlab field area, much of this reduction is the result of the family-planning programme and social changes in society rather than improvements in delivery-care. That is, first births are occurring at a somewhat later age, and women are having fewer high-risk pregnancies and fewer total numbers of children. Although more hospitals have emergency obstetric facilities, many of these are not functional because they are not staffed consistently. Costs for delivery-care, especially for caesarean deliveries, are not affordable for most families. Most deliveries still take place in the home and are assisted by unskilled birth attendants. Estimates found a very high level of unmet obstetric need in most upazilas, and the unmet need increased substantially with distance from the emergency obstetric facility.

Since reducing maternal mortality is a priority for the Ministry of Health and Family Welfare (MoHFW), there are plans to train many more skilled birth attendants. However, according to calculations of ICDDR, B, the current plans will not meet the demand for skilled birth attendants during the coming decade, especially if the current plan of emphasizing deliveries at home is continued. An alternative strategy of facility-based deliveries in which practice of skilled birth attendants as a group with close connections to upazila-level emergency obstetrical care is more likely to rapidly meet the needs of the mothers of Bangladesh.

Potentially-serious complications of delivery are not always easy to detect, since they may be prolongation of a normal-delivery. Providers, families, and mothers must learn how to detect these complications early so that medical help can be obtained quickly. Mortality from postpartum haemorrhage, one of the most common lethal complications, may be reduced through the use of active management of the third stage of labour, including the use of misoprostol, and a trial of this is now ongoing at ICDDR, B.

Infectious diseases remain as major problems in Bangladesh. Although mortality from diarrhoeal diseases has decreased remarkably, mortality from pneumonia has not improved significantly. The success in the management of diarrhoea is a major achievement for Bangladesh and for the partners that facilitated this, including the MoHFW, ICDDR, B, BRAC, USAID, and the Social Marketing Company. Nearly all families know and use ORS appropriately—a situation much different from other developing countries. New vaccines for rotavirus are now available, and operations research is needed to determine whether these are the vaccines which Bangladesh can afford. Studies of the effectiveness of rotavirus vaccine planned for Bangladesh will guide decisions of Global Alliance for Vaccines and Immunization regarding financial support for the vaccine in eligible countries. Cholera vaccine is another one which is needed in Bangladesh. As newer formulations of the oral vaccine become less expensive and more convenient, this vaccine should be introduced; however, due to need for repeated periodic dosing schedule and older target age-groups, a strategy other than EPI will be needed—perhaps through a social market approach and distribution by an over-the-counter product. Implementing vaccines for rotavirus and cholera could reduce rates of severe, life-threatening diarrhoea by more than 50%. For ICDDR, B, this could avert more than 50,000 hospitalizations per year. Improvements in water and sanitation are also needed, and there are many opportunities for scaling up community-based measures, such as handwashing and point-of-use water purification, rather than waiting for large-scale engineering projects to provide water and sanitation.

In contrast to diarrhoea where progress has been excellent, new strategies to reduce mortality due to pneumonia are urgently needed. These new strategies will likely include the inclusion of vaccines for *Haemophilus influenzae* type b (Hib) and eventually *Streptococcus pneumoniae* in the EPI, as well scaling up zinc therapy for diarrhoea (to reduce future episodes of pneumonia) and improving case management. Zinc therapy, although targeted to the treatment of diarrhoea, may have its greatest benefit in reducing rates of severe pneumonia among those children who receive it consistently for diarrhoea. Children who have frequent bouts of diarrhoea often may be the same children who are most likely to suffer from fatal pneumonia, and the zinc therapy can reduce this probability significantly. Zinc has an independent effect on pneumonia, and further studies are needed to better understand how to use zinc to reduce morbidity and mortality due to pneumonia. Severe pneumonia is such a common infection; there are probably neither a sufficient number of hospital beds to accommodate the needs, nor are there sufficient resources to pay for this hospital treatment. Thus, new strategies are needed to provide adequate care for these large numbers of patients. A day-care model is being evaluated and appears to be as successful as hospitalization for nearly all patients.

Tuberculosis is another critical infectious disease which needs additional resources. Fortunately, the national programme is effectively partnering with several NGOs and ICDDR, B, to improve the quality of services through the directly-observed therapy short-course strategy. However, additional resources and strategies are needed to identify cases earlier, before patients have transmitted their infection to others. The predominance of males with tuberculosis may offer insights into this infection; this may partially be explained by smoking which is a risk factor for tuberculosis, but other factors need to be explored. Antibiotic resistance in tuberculosis is currently at a low level, but threatens to become more common. Clearly, resistance needs monitoring. Rapid control of tuberculosis for Bangladesh is especially critical, since the threat of HIV/AIDS will greatly complicate future efforts.

The prevalence of HIV/AIDS infection continues to be low in Bangladesh. Still, high-risk behaviours are very common in the ‘most-at-risk’ groups and in the general population. Rates of HIV prevalence in one group of injecting drug users already have reached epidemic levels, and we suspect that the infection will likely spread to other groups of injecting drug users and into the population of sex workers and then to the general population. A computer model for the likely course of the epidemic for Dhaka city was developed and adds emphasis to the need for preventive measures early in the epidemic. Considerable sums have been allocated for HIV/AIDS-prevention activities from the World Bank, United States Agency for International Development, Global Fund for AIDS, TB and Malaria, and UK Department for International Development, and these have stimulated many projects. A constraint for these activities, however, has been the inconsistent funding for many specific programmes, making continuity difficult for the implementers and leaving gaps in the overall strategy.

The situation with nutrition has shown a gradual overall improvement, but progress has been relatively slow. Malnutrition rates remain high. Of the 3-5 years old children, about 50% are stunted, 55% are underweight, and about 12% are wasted. These trends have been improving, but very slowly. Some key interventions needed to improve the nutritional status (and mortality) include renewed efforts to encourage exclusive breastfeeding for the first six months and improved complementary feeding after six months. Nationally, rates of exclusive breastfeeding have not improved, although several small-scale interventions showed that this behaviour change is possible. Complementary feeding needs considerable efforts both in terms of the quality of the food provided and the feeding behaviours of the families.

Success in improving micronutrient deficiency has been mixed. The vitamin A programme has been extremely successful, and its coverage improved remarkably when it was integrated with the national immunization days. Clinical vitamin A deficiency is now rarely seen, although serum levels remain low, especially in children aged over five years who do not receive vitamin A through the national programme. Continued efforts are needed to insure that vitamin A intake is sufficient through supplements and fortification where possible. Iodine fortification has been relatively less successful. Despite a national strategy to include iodine in all salts marketed in the country, universal coverage has not occurred, and efforts are needed to correct this. Anaemia is common in Bangladesh and has shown no improvement over time. In fact, rates of anaemia now are very similar to rates seen for the last several decades. Although iron deficiency is also common, it is an over-simplification to conclude that most anaemia in Bangladesh is caused by iron deficiency. There appears to be other causes as well, and research is needed to determine the relative contribution of these causes and to validate the effectiveness of the potential interventions before we can expect major improvements.

Zinc is the exciting new intervention in which Bangladesh is a leader. Although zinc deficiency has long been recognized in children in many deve-loping countries, especially in Asia, the incredible health benefits of providing zinc to children are a recent discovery. Since zinc is not stored in the body like vitamin A, it was thought that one would have to provide zinc daily, and this seemed to be logistically impossible. In studies showing its benefits in the treatment of diarrhoea, the long-term bene- fit of such consistent treatment was documented with reductions in the incidence of diarrhoea, the incidence of pneumonia, and the overall probabili- ty of death among children aged less than five years. The clear health benefits and the safety of this treatment led to the recommendations from the World Health Organization and United Nations Children's Fund that all children with diarrhoea should receive a 10- to 14-day course of zinc. ICDDR, B has now a project to scale up this recommendation; however, research on how to maximize the impact of zinc continues. In the future, zinc may also be recommended with each EPI visit and in the treatment of pneumonia. Additionally, studies are ongoing to supplement foods with zinc on a routine basis so that children do not become zinc-deficient.

Domestic violence is a public-health topic that is rarely discussed, yet it must be included in this volume because of its crucial importance to the women and families of Bangladesh. The Government of Bangladesh has made elimination of violence among intimate partners a priority, and a recent multicountry study carried out by the WHO has documented just how common and damaging this problem is, not just in Bangladesh, but globally. Over half of adult women in Bangladesh are victims of violence from intimate partners, and these women and their children suffer many health and emotional consequences from this abuse. Unfortunately, most abuse is hidden, since families do not want to bring shame on themselves, but interventions are desperately needed that will stop the violence and make this behaviour unacceptable.

Although public-health services are intended to be equitable, monitoring the programmes to determine if they are reaching the poor is not straightforward. Some improvements in monitoring systems have been developed, but still need to be validated and tested on a larger scale. Some principles of equity are clear. Preventive programmes, e.g. vaccines, vitamin A, and zinc, are inherently more equitable, since they can be provided to all strata of society. Interventions that depend on treatment, by contrast, will almost always be less equitable, since they require access and connections, and out-of-pocket costs for patients. The new tools are intended to be useful for programme managers to periodically monitor whether their programme is pro-poor. If it is not pro-poor, the manager is then able to make adjustments.

The role of research and surveillance in achieving the MDGs must be highlighted. The information in this volume are possible only because of ongoing surveillance and occasional surveys. Without this information, many health issues would go unrecognized, and progress towards improvements would be impossible to monitor. Without research, we would have no new technologies or new approaches to apply in achieving the MDGs. Perhaps equally important is the operations and health systems research. Although the various *Lancet* issues advocating for achieving the MDGs make the point that we can achieve the MDGs using tools that are available today (implying that further research is not needed), I would argue that research is critical so we can learn how to use these tools to actually make a difference. This type of research may not win Nobel prizes for basic discoveries, but this is the type of research that will lead to control of diseases. An illustration of the importance of this kind of downstream research was the control of smallpox which was not achieved simply with the vaccine which had been available for hundreds of years, but rather with a new strategy for how to vaccinate populations at risk. It used the technology (the vaccine) but without the new strategy (case detection and targeted vaccinations around the case), smallpox would never have been eradicated.

## CONCLUSION

Achieving MDG 1, 4, 5, and 6 will be a challenge for Bangladesh. This will require a coordinated effort to improving health services for mothers and children, improving nutritional status, reducing the burden of infectious diseases, and using modern technologies in a cost-effective manner. This volume is intended to stimulate discussion on how to improve these services most effectively.

